# Clinical relevance of *LINC00152* and its variants in western Chinese tuberculosis patients

**DOI:** 10.18632/oncotarget.23297

**Published:** 2017-12-14

**Authors:** Jing Li, Lijuan Wu, Weihua Guo, Juli Chen, Xuejiao Hu, Minjin Wang, Zhenzhen Zhao, Binwu Ying

**Affiliations:** ^1^ Department of Clinical Laboratory, Central Hospital of Panzhihua City, Panzhihua, Sichuan 617067, P. R. China; ^2^ Department of Laboratory Medicine, West China Hospital, Sichuan University, Chengdu, Sichuan 610041, P. R. China

**Keywords:** tuberculosis, LINC00152, genetic variants, susceptibility, adverse drug reaction

## Abstract

Recent studies indicate that the long intergenic non-coding RNA *LINC00152* plays crucial roles in various human diseases. Here, we investigated whether levels of *LINC00152* or its genetic variants correlate with the clinical features of tuberculosis (TB) in western Chinese patients. We genotyped the single nucleotide polymorphism *LINC00152* rs80292941 in 476 TB patients and 475 healthy subjects using a custom-by-design 48-Plex SNPscan Kit, and measured relative levels of *LINC00152* using RT-qPCR. We observed that *LINC00152* levels were lower in TB patients than controls. Moreover, rs80292941 TT genotype carriers had the lowest *LINC00152* levels among TB patients, and rs80292941 AA genotype carriers are more likely to suffer from hepatotoxicity induced by antituberculosis therapy [OR = 3.97, 95% = 1.53-10.13, *p* = 0.002]. Our findings strongly suggest that *LINC00152* may promote TB progression and highlight rs80292941 single nucleotide polymorphism as a novel predisposition marker for antituberculosis drug-induced hepatotoxicity.

## INTRODUCTION

Human tuberculosis (TB) resulting from Mycobacterium tuberculosis (MTB) infection remains a major public health threat all worldwide, with an estimated 10.4 million new cases and 1.7 million deaths occurring in 2016 [1]. TB control efforts face many major challenges, such as side effects from anti-TB chemotherapy, an increase in multidrug-resistant TB (MDR-TB) strains, and the prevalence of co-infection with human immunodeficiency virus (HIV) [2]. In spite of recent advances that have improved the diagnosis and treatment of TB, the molecular mechanisms underlying this disease remain unclear. There is thus a dire need to further investigate the specific molecules and signaling pathways involved in MTB infection and to develop novel molecular markers for the identification and treatment of human TB.

The Encyclopedia of DNA Elements (ENCODE) and Human Genome Project show that over 75% of the human genome is transcribed into primary transcripts, producing a broad range of non-coding RNA [3]. Long non-coding RNAs (lncRNAs) are RNA transcripts of more than 200 nucleotides that have a defective open reading frame [4] and participate in diverse biological processes [5, 6] underlying human disorders [7–9], especially malignant tumors. A newly delineated lncRNA, long intergenic non-coding RNA 00152 (*LINC00152*), is upregulated in human gastric tissues relative to adjacent cancer-free tissues [10] and might affect cell proliferation, cell cycle arrest, apoptosis, epithelial to mesenchymal transition (EMT), cell migration and invasion in gastric cancer [11]. Multiple studies support the oncogenic role of *LINC00152* in several human carcinomas [12–15]. Taken together, these data suggest that lncRNA *LINC00152* could serve as a new biomarker for the detection and treatment of multiple cancers. Deng *et al* [13] reported that hepatitis B virus (HBV) X-protein (HBx)-induced *LINC00152* production could contribute to cancer progression in hepatocellular carcinoma associated with HBV. According to Wang *et al* [14], *LINC00152* promotes renal cell carcinoma progression by acting as a microRNA-205 sponge and by epigenetically inhibiting P16 expression. Hu *et al* [12] showed that *LINC00152* levels in peripheral circulation might be early predictors of esophageal squamous cell carcinoma (ESCC).

An increasing number of studies suggest that lncRNAs also promote human TB infection [16–19]. Wang *et al* [16] showed that lncRNA-CD244 suppresses IFN-γ/TNF-α expression by mediating H3K27 trimethylation in infg/tnfa loci of CD8+ T cells. Another study [19] revealed distinct expression signatures for lncRNAs in macrophages after infection with H37Ra or H37Rv, showing that lncRNAs MIR3945HG V1 and MIR3945HG V1 could be used as diagnostic biomarkers for TB, with excellent area under the curve (AUC) values of 0.925 and 0.956, respectively. However, the expression pattern, molecular function, and clinical relevance of *LINC00152* in TB remains unexplored. Genetic association is the main approach used to identify whether genomic variant loci correlate with TB susceptibility. In addition to genetic variations within protein-coding genes, several single nucleotide polymorphisms (SNPs) located in non-coding genomic sequences have also been studied. We previously showed that rs12477677 within lncRNA AC0797767.4 is linked to a diminished predisposition to pulmonary tuberculosis (PTB) while potentially influencing clinical phenotypes of active TB [20]. Consequently, screening for SNPs from the large collection of human genomic protein non-coding regions is a promising strategy for the identification of TB susceptibility loci.

China is experiencing a serious TB epidemic. In western China, the annual incidence rate of PTB (up to 695 cases per 100,000 individuals) is remarkably higher than in other areas [21]. Therefore, whether *LINC00152* and its genetic variations promote active TB is an attractive question deserving of further investigation. We thus carried out a case-controlled study of 476 TB cases and 475 healthy controls among western Chinese inhabitants to determine whether levels of *LINC00152* and rs80292941 correlate with active TB disease risk and clinical features. Moreover, we performed a prospective follow-up analysis to investigate whether *LINC00152* and its polymorphism rs80292941 affect the response of patients to anti-TB chemotherapy.

## RESULTS

### Study subject demographic traits and clinical information

Demographic and clinical features and quantitative inspection results of the cases and controls are shown in Table 1. We generally found no statistically significant differences in age or gender between the cases and controls, showing that both groups were frequency-matched with respect to age and sex. When comparing these laboratory parameters at baseline, TB patients showed distinct biochemical parameters and hematological blood patterns relative to those of healthy individuals, and cases with active TB presented elevated white blood cell and platelet counts as well as alanine transaminase (ALT) and aspartate transaminase (AST) levels compared to those of controls (p < 0.001 for all). TB patients exhibited lower hemoglobin (Hb), hematocrit (Hct), and erythrocyte levels (p < 0.001 for all) than healthy participants. As expected, the TB case group showed an obvious increase in C-reactive protein (CRP) and erythrocyte sedimentation rate (ESR) levels relative to those of the control group (p < 0.001 for both).

**Table 1:** Demographic/clinical characteristics of the study participants

Features	TB (n = 476)	HC (n = 475)	*P*
**General information**			
Age, mean ± SD (years)	41.47 ± 19.29	42.60 ± 13.27	0.291
Male/female	289/187	270/205	0.225
**Laboratory parameters before anti-TB**			
Erythrocyte (×10^12^/L)	4.20 ± 0.82	4.80 ± 0.48	**<0.001**
Hematocrit (%)	37.00 (32.00−41.00)	45.00 (40.00−48.00)	**<0.001**
Hemoglobin (g/L)	118.35 ± 24.22	145.59 ± 15.16	**<0.001**
Platelets (×10^9^/L)	251.31 ± 117.75	170.92 ± 46.89	**<0.001**
ALT (IU/L)	22.00 (16.00−32.00)	11.00 (7.00−16.75)	**<0.001**
AST (IU/L)	18.00 (14.00−23.00)	23.00 (19.00−29.00)	**<0.001**
CRP (mg/L)	19.85 (6.04−67.13)	5.49 (1.70−18.42)	**<0.001**
ESR (mm/h)	48.00 (22.00−78.50)	20.38 (8.43−56.61)	**<0.001**
**TB clinical subtype n (%)**			
PTB	283 (59.45)		
EPTB	109 (22.90)		
PTB & EPTB	84 (17.65)		
**TB clinical condition n (%)**			
De novo	222 (46.64)		
Retreated	254 (53.36)		
**TB-related systemic symptoms n (%)**			
Fever	212(44.54)		
Loss weight	149(31.30)		
Night sweat	120(25.21)		
Poor appetite	156(32.77)		
Fatigue	99(20.80)		
**Local chest symptoms n (%)**			
cough	307(64.50)		
hemoptysis	72(15.12)		
Chest pain	161(33.82)		
Dyspnea	24(5.04)		
**TB etiological evidence**			
TB-DNA positive/negative	173/136 (55.99)		
Smear positive/negative	140/446 (31.39)		
Culture positive/negative	23/93 (19.83)		
**CT scan at baseline n (%)**			
Bilateral lung damage	244/360 (67.78)		
Unilateral lung damage	103/360(28.33)		
None lung damage	14/360 (3.89)		
**TB-related adverse drug reactions n (%)**			
Anemia	73 (15.33)		
Leukopenia	80 (16.80)		
Thrombocytopenia	31 (6.51)		
Drug-induced hepatotoxicity	73 (15.33)		
Chronic kidney damage	7 (1.47)		

Annotation: TB = tuberculosis; HC = healthy controls; PTB = pulmonary tuberculosis; EPTB = extra-pulmonary tuberculosis; PTB & EPTB = pulmonary tuberculosis combined with extra-pulmonary tuberculosis.

As is shown in Table 1, we also reviewed a range of clinical and laboratory indexes correlated with TB disease, including clinical subtypes and conditions, etiological evidence, symptoms, and computed tomography (CT) results. We recruited 476 patients with active TB, of which 283 exhibited PTB (PTB, 59.45%), 109 exhibited extra-pulmonary tuberculosis (EPTB, 22.90%), and 84 exhibited PTB combined with EPTB (PTB&EPTB, 17.65%). Among all cases, 46.64% (222/476) had de novo TB, and 53.36% (254/476) had undergone anti-TB treatment for at least one month. In total, 55.99% (173/309) of the TB-DNA positive rate was higher than positive rates for microscope smears and bacterial cultures [31.39% (140/446), 19.83 (23/93), separately]. In our study, only a relatively small number (n=93) of the patients with TB had undergone TB culture examination. Regarding clinical symptoms, patients (44.54%) mainly complained of fever, and a majority of individuals (64.50%) suffered from a cough. Of all of the 476 cases with TB, 360 subjects had received a CT scan on admission, of which 67.7% (244/360) had experienced bilateral lung damage, 28.33% (102/360) had developed a unilateral lung lesion, and only 3.89% (14/360) had not experienced lung injury.

For our case cohort, 272 TB patients received 2HREZ/4-7HRE [a regimen of isoniazid (H), rifampicin (R), ethambutol (E) and pyrazinamide (Z) for 2 months, followed by 4-7 months of HRE] while the remaining 204 received 2HREZS/2-5HRE (S means streptomycin). All recruited TB patients underwent three routine laboratory tests at baseline before receiving anti-TB therapy. These patients were then subjected to monthly laboratory examination. Follow-up laboratory examination results for all of the patients are as follows: HB-valley: 116.85 ± 25.02 g/L, PLT-valley: 234.75 ± 114.32 × 10^9^/L, WBC-valley: 5.06 (3.86-6.43) × 10^9^/L, ALT-peak: 28.50 (15.25-52.75), AST-peak: 29.00 (20.00-53.00), Creatinine-peak: 62.85 (50.00-77.00). Leukopenia (16.80%, 80/476) was found to be the most common hematological adverse event involving RIF and INH use followed by anemia (15.33%, 73/476) and drug-induced hepatotoxicity (15.33%, 73/476). Only seven patients had suffered from chronic kidney damage (CKD) associated with anti-tuberculosis drugs (ATDs).

### SNP rs80292941 association with TB susceptibility

#### Genotyping results

The candidate SNP rs80292941 within the *LINC00152* gene sequence was genotyped successfully in all 476 patients with TB disease and in the 475 control individuals. Genotype distributions of polymorphism rs80292941 in the control group were in compliance with Hardy-Weinberg equilibrium (HWE) (p-value = 0.658), and the minor allele frequency (MAF) was 0.114.

#### Candidate rs80292941 association analysis

Allele and genotypic distributions of the rs80292941 locus between the TB cases and healthy populations are shown in Table 2. Proportions of minor allele T in rs80292941 loci in the case and control groups were 11.13% and 11.68%, respectively. The allelic distribution of this locus was largely analogous between the two groups with a p-value of 0.706 (OR = 0.95, 95% CI = 0.71 - 1.26). For the cases with active TB, the frequencies of genotypes TT, AT and AA were 2.10%, 18.07%, and 77.68%, respectively, and TT, AT and AA genotype rates of the controls were 1.06%, 21.26%, and 77.68%, respectively, with a statistically significant p-value of 0.220. To further investigate genotype distribution differences between these 2 groups, we performed a genetic model analysis (including a dominant and recessive model). Our analyses failed to find any statistically significant differences in dominant and recessive genetic patterns between the cases and controls (p > 0.05 for all as shown in Table 2).

**Table 2:** SNP rs80292941 in relation to susceptibility to TB

SNP variations	Case (n %)	Control (n %)	*P*	95% CI
Allele				
T	106 (11.13)	111 (11.68)	0.706	0.95 (0.71 − 1.26)
A	846 (88.87)	839 (88.32)		
Genotype				
TT	10 (2.10)	5 (1.06)	0.220	
AT	86 (18.07)	101 (21.26)		
AA	380 (79.83)	369 (77.68)		
Dominant model				
TT+AT	96 (20.17)	106 (22.32)	0.418	0.88 (0.64 − 1.20)
AA	380 (79.83)	369 (77.68)		
Recessive model				
TT	10 (2.10)	5 (1.05)	0.195	2.01(0.68 − 5.95)
AT+AA	466 (97.90)	470 (9.95)		

Annotation: *p*-value was calculated by Chi-square test.

As several given SNPs may be correlated with the specific TB subtype reported in previous studies [20], we conducted multiple subgroup analyses. We classified all TB cases into three subgroups based on TB clinical subtypes: 283 PTB patients, 109 EPTB patients, and 84 patients with PTB&EPTB. According to the TB clinical treatment condition, all cases were stratified as two subsets: 222 newly diagnosed TB cases and 254 re-treated cases. Unfortunately, we did not find any positive association between these TB subsets and healthy controls (data not shown).

#### Relationship between rs80299241 genotypes and TB phenotypes

We comprehensively assessed the potential correlation between different rs80292941 genotypes and TB disease manifestation. Several common indexes of active TB disorder were evaluated (*i.e.*, TB-related systemic and local symptoms, complete blood cell test results, TB etiological analysis results, ESR, CRP levels, and lung lesions observed from chest CT scans). Due to infrequent proportions of minor homozygous genotypes (TT) found in rs80292941, we drew comparisons based on the dominant pattern found (AA vs. AT+TT). For the rs80292941 locus, none of the analyzed variations correlated with genotype distributions (Table 3). We only found that patients with the minor allele (T allele) and with both homozygotes and heterozygotes appeared to suffer less from night sweats than those with the homozygous major allele (AA), although the related p-value was not statistically significant (p = 0.064, Table 3).

**Table 3:** Correlation between rs80292941 polymorphism and TB phenotype traits

Characterizations	AA (N=380)	AT + TT (N=96)	*P*
**Clinical phenotypes n (%)**			
Systemic symptoms			
Fever	170 (80.2)	42 (43.75)	0.862
Weight loss	128 (40.76)	32 (33.33)	0.948
Night sweat	111 (32.48)	19 (19.79)	0.064
Poor appetite	136 (38.22)	30 (31.25)	0.404
Fatigue	92 (38.22)	18 (18.75)	0.257
Local chest symptoms			
cough	242 (57.76)	65 (67.71)	0.462
hemoptysis	59 (14.29)	13 (13.54)	0.628
chest pain	68 (34.16)	17 (17.71)	0.966
dyspnea	20 (7.45)	4 (4.17)	0.859
**Laboratory traits**			
Full blood cell counts			
Erythrocyte (×10^12^/L)	4.18 ± 0.84	4.28 ± 0.76	0.295
Hematocrit (%)	37.00 (32.00−41.00)	38.00 (32.00−42.00)	0.471
Hemoglobin (g/L)	118.10 ± 24.40	119.36 ± 23.56	0.647
Platelets (×10^9^/L)	252.18 ± 118.75	247.86 ± 114.22	0.749
Leucocytes (×10^9^/L)	6.49 (4.80−8.90)	6.29 (4.73−8.63)	0.362
Neutrophils (×10^9^/L)	4.64 (3.15−6.58)	4.19 (3.01−6.26)	0.172
Lymphocytes (×10^9^/L)	1.07 (0.75−1.52)	1.25 (0.79−1.71)	0.152
Monocytes (×10^9^/L)	0.46 (0.31−0.67)	0.45 (0.29−0.62)	0.407
Routine TB markers			
CRP (mg/L)	22.35 (7.63−76.85)	19.35 (6.81−69.25)	0.214
ESR (mm/h)	45.00 (23.00−73.00)	43.00 (22.00−74.00)	0.824
TB etiological assays			
Positive TB-DNA	136 (55.28)	37 (58.73)	0.623
Positive smear	111 (31.18)	29 (32.22)	0.849
**CT scanning**			
Bilateral lung lesion	191 (66.55)	55 (75.34)	0.204
Unilateral lung lesion	86 (29.97)	17 (23.29)	

### Comprehensive analysis of changes in *LINC00152* expression levels in TB cases

#### Expressive levels of LINC00152 between cases and controls

**Figure 1:**
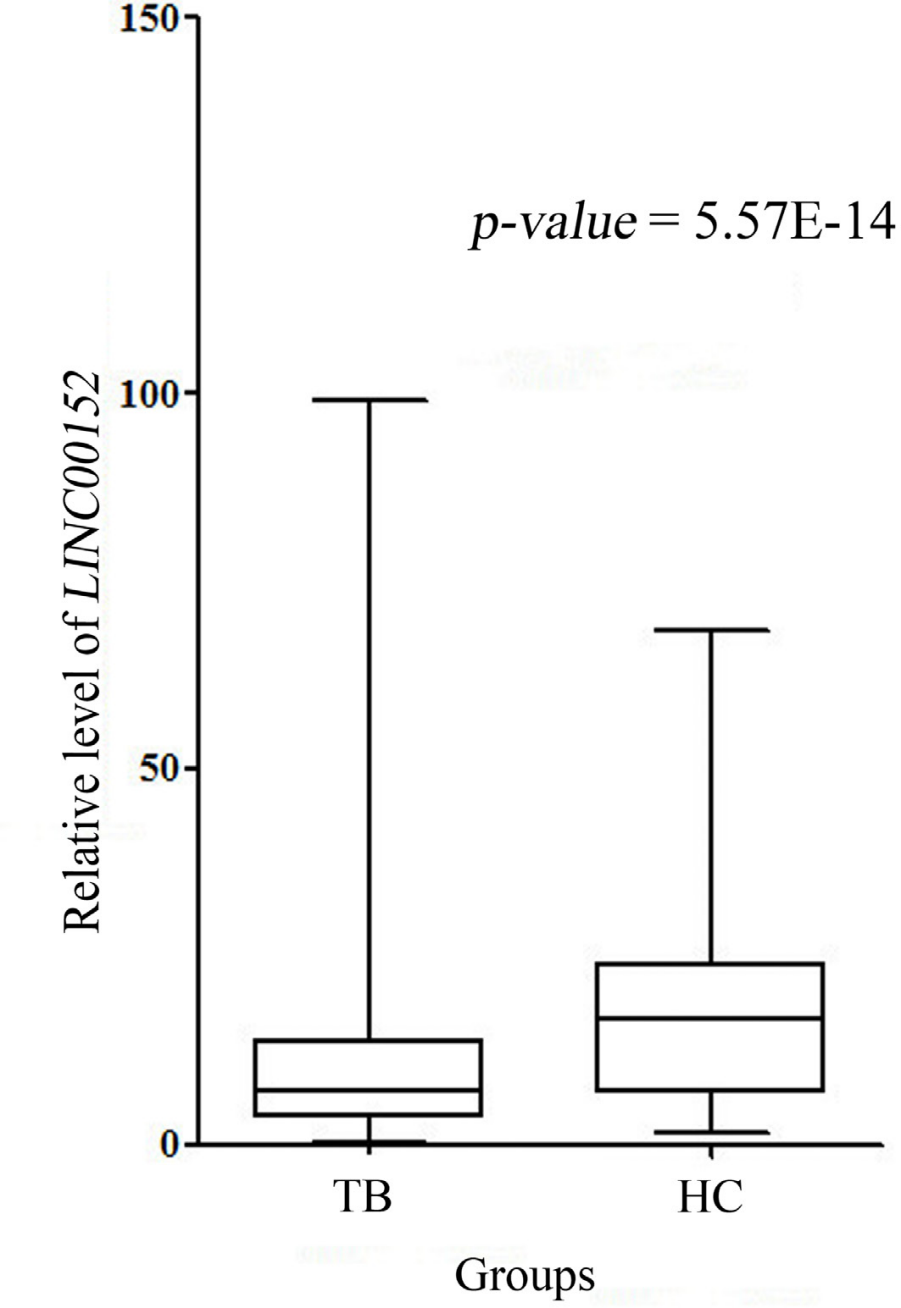
*LINC00152* relative expression in cases and controls Note: There was abundant reduction in *LINC00152* expression in cases compared with controls (p < 0.001).

Our real-time PCR quantitative results showed that the case group had lower *LINC00152* relative levels normalized to the experimental endogenous control, compared to those of the control group [cases: 7.45 (3.60-15.51); controls: 16.77 (6.81-24.05), p < 0.001] (Table 4, Figure 1), implying that *LINC00152* might modulate the development and progression of TB.

**Table 4:** Relative expression level of *LINC00152* in cases and controls

*LINC00152*	TB (n = 476)	HC (n = 475)	*P*
Relative level (×10^4^)	7.45 (3.60−15.51)	16.77 (6.81−24.05)	**<0.001**

Annotation: The data was displayed as median (interquartile range, IQR) and was compared with Mann-Whitney U method.

#### Investigation of the role of SNP rs80292941 in LINC00152 expression

Using lncRNA SNP bioinformatics software [22] (http://bioinfo.life.hust.edu.cn/lncRNASNP/), we found that candidate genetic variation rs80292941 might influence the binding of lncRNA *LINC00152* to several microRNAs (miRNAs) including both gains and losses. We hypothesized that rs80292941 is indirectly associated with *LINC00152* expression. To test this, we evaluated the effects of rs80292941 on *LINC00152* levels. Relative levels of *LINC00152* derived from all 476 TB cases and 475 controls were compared across the three different SNP genotypes. We found substantial discrepancies in levels of *LINC00152* among different rs80292941 genotypes for the case groups [TT: 2.79 (1.86-6.29) versus. AT: 9.38 (3.99-21.01) versus. AA: 7.45 (3.75-15.22), p = 0.018, Table 5], among which homozygous minor allele (TT) carriers expressed the lowest *LINC00152* transcript levels. The *LINC00152* levels of three different genotypes were comparable for the healthy control group (TT: 15.75 (5.45-24.98) versus. AT: 17.36 (6.54-24.56) versus. AA: 16.77 (6.81-24.05), p = 0.973, Table 5). For cases involving TB infection, we surprisingly found that *LINC00152* levels had remarkably decreased in all three rs80292941 genotypes relative to those of healthy subjects (p = 0.040 for TT, p = 0.008 for AT, p < 0.001 for AA as shown in Table 5). Interestingly, Table 5 shows that TB patients with TT genotypes may show the greatest decline in *LINC00152* levels relative to those of the other two genotypes.

**Table 5:** Relative expression level of *LINC00152* in different rs0292941 genotypes between cases and controls

Genotypes	Cases	Controls	*P* between cases and controls
TT genotype	2.79 (1.86−6.29)	15.75 (5.45−24.98)	**0.040**
AT genotype	9.38 (3.99−21.01)	17.36 (6.54−24.56)	**0.008**
AA genotype	7.45 (3.75−15.22)	16.77 (6.81−24.05)	**<0.001**

Annotation: *p* = 0.018 and 0.973 for three genotypes in case group and control group, respectively.

### *LINC00152* and rs80292941 associations with adverse drug reactions related to TB treatment

It was previously reported that upregulated *LINC00152* is positively associated with resistance to oxaliplatin chemotherapy in colon cancer [23]. In this study, we innovatively assessed whether the *LINC00152* and its polymorphism rs80292941 correlated with adverse drug reactions induced by TB chemotherapy. We analyzed common TB-related adverse reactions in our datasets involving cases of anemia, leukopenia, thrombocytopenia, hepatotoxicity, and CKD, which were dependent on positive laboratory examination results and clinical manifestations when possible. We failed to observe any associations between *LINC00152* relative levels and the occurrence of five adverse drug responses from TB chemotherapy (Table 6). However, the TB non-susceptibility locus, the dominant model of rs80292941 [AA/(AT+TT)], is closely associated with the presence of drug-induced hepatotoxicity in the dominant model. Patients carrying homozygous for the A allele (AA genotype) were more likely to bear hepatotoxicity resulting from anti-TB therapy than T allele carriers (AA: 17.89% vs. AT + TT: 5.21%) with an estimated p = 0.002 (OR = 3.97, 95% CI = 1.53-10.13 as shown in Table 7).

**Table 6:** Relationships between *LINC00152* transcript and TB-related drug adverse responses

Drug adverse reactions	*LINC00152* levels	*P*
**Anemia n (%)**		0.348
Presence (73, 15.33)	9.63 (3.52−13.72)	
Absence (403, 84.67)	7.56 (3.69−17.63)	
**Leukopenia n (%)**		0.769
Presence (80, 16.80)	7.20 (3.91−14.80)	
Absence (396, 83.20)	7.56 (3.55−15.97)	
**Thrombocytopenia n (%)**		0.775
Presence (31, 6.51)	6.72 (2.87−30.01)	
Absence (445, 93.49)	7.56 (3.73−15.22)	
**Drug-induced hepatotoxicity n (%)**		0.602
Presence (73, 15.33)	6.35 (3.56−15.06)	
Absence (403, 84.67)	7.61 (3.65−15.70)	
**Chronic kidney damage n (%)**		0.419
Presence (7, 1.47)	5.40 (3.45−12.42)	
Absence (469, 98.53)	7.50 (3.60−15.81)	

**Table 7:** Relationships between rs80292941 and TB-related drug adverse responses in the dominant model

Drug adverse reactions	AA (N = 380)	AT + TT (N = 96)	*P*	OR (95% CI)
**Anemia n (%)**	57 (15.00)	16 (16.67)	0.686	0.88 (0.48−1.62)
**Leukopenia n (%)**	62 (16.32)	18 (18.75)	0.569	0.84 (0.47−1.51)
**Thrombocytopenia n (%)**	25 (6.58)	6 (6.25)	0.907	1.06 (0.42−2.65)
**Drug-induced hepatotoxicity n (%)**	**68 (17.89)**	**5 (5.21)**	**0.002**	**3.97 (1.53−10.13)**
**Chronic kidney damage n (%)**	4 (1.05)	3 (3.13)	0.149	0.33 (0.73−1.50)

## DISCUSSION

Long intergenic non-coding RNA 00152, *LINC00152*, is situated in chromosome 2p11.2 and has a transcript with 828 nucleotides. Here, we explored whether expression of *LINC00152* and its variant rs80292941 correlates with TB predisposition, disease presentation, or adverse reactions to anti-TB drugs. We found that *LINC00152* expression is markedly decreased in cases of TB infection and that different rs80292941 genotypes modulate *LINC00152* expression. Furthermore, we found that the rs80292941 locus correlates with increased hepatotoxicity levels after anti-TB therapy.

In our study, polymorphism rs80292941 in the *LINC00152* gene did not increase susceptibility to TB in western Chinese. However, for patients with TB, we found significant differences in *LINC00152* rs80292941 levels for three genotype groups (TT vs. AT vs. AA genotype), with homozygous TT genotype carriers showing the lowest levels. In addition, patients with the TT genotype may contribute most to the downregulation of *LINC00152* for the case group when compared to the healthy controls. SNP rs80292941 located in the untranslated region of the *LINC00152* gene may affect the interaction between *LINC00152* and many miRNAs and may subsequently dysregulate *LINC00152* transcript expression. Supplementary Figure 1 shows the miRNA binding locus within rs80292941 in the presence of the T allele. On the other hand, in the presence of the A allele, the binding locus is lost (Supplementary Figure 2). MiRNA is involved in the function of RNA-induced silencing complexes, which play a role in degrading complimentary target mRNAs or blocking their translation. As for noncoding RNA, Zhang *et al.* demonstrated that miR-376c-3p negatively regulated the expression of *LINC00152* in colorectal cancer cells, restricting cell viability and stimulating cell apoptosis [24]. Therefore, here we hypothesize that some functional miRNAs might bind rs80292941 TT, thereby lowering *LINC00152* expression, which should be further tested with functional assays.

**Figure 2:**
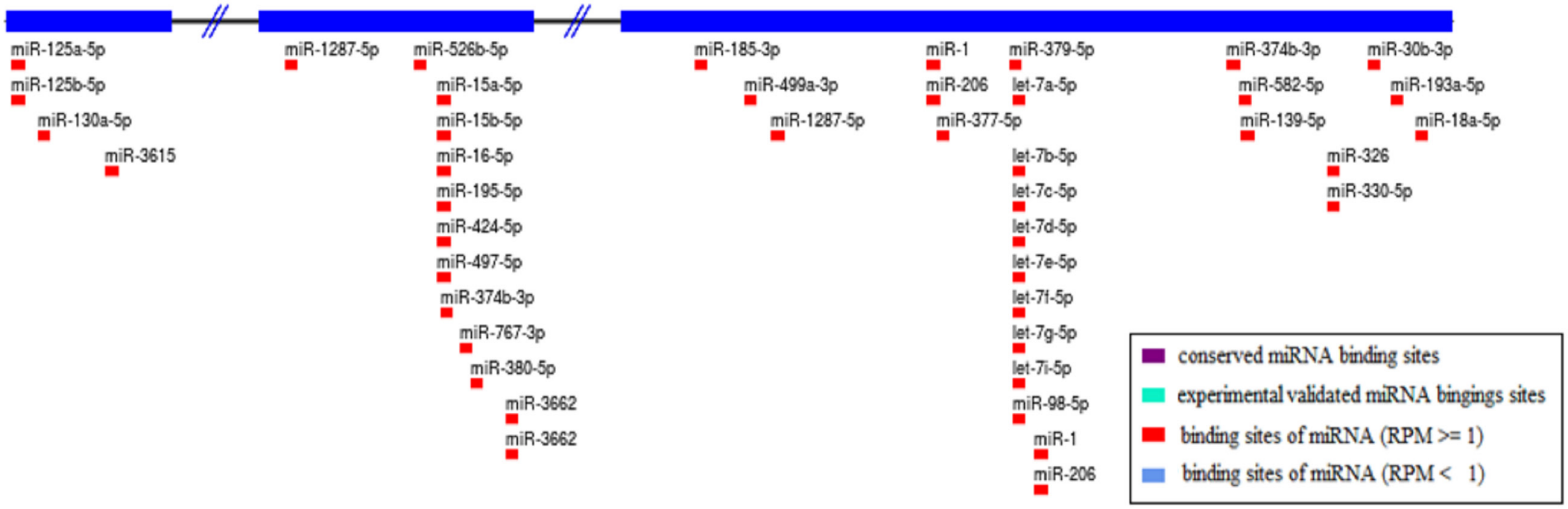
Prediction of miRNAs targeting *LINC00152* Note: A total of 38 kinds of miRNAs were obtained through the lncRNA SNP database.

We found *LINC00152* to be substantially downregulated in TB cases relative to controls. Compelling data from previous studies have generally shown that lncRNA *LINC00152* is upregulated in many types of human malignant cancers (including gastric cancer, colorectal cancer, etc.) and accelerates carcinoma progression and migration through diverse molecular mechanisms. For example, Cai *et al* [25] showed that *LINC00152* overexpression induced by transcription factor specificity protein 1 (SP1) might be involved in the phosphatidylinositol 3-kinase (PI3K)/AKT pathway and act as an oncogene in gallbladder cancer (GBC). Further mechanistic analyses also suggested that *LINC00152* acts as a competing endogenous RNA (ceRNA) to sponge miR-138 and subsequently inhibit HIF-1α (hypoxia inducible factor-1α) expression, ultimately contributing to GBC metastasis and epithelial-mesenchymal transition [26]. Recently, more attention has been dedicated to how lncRNAs functioning as ceRNAs are involved in biological development and human disorders. We speculate that *LINC00152* might promote TB infection by interacting with miRNAs markers. From the lncRNA SNP database, we identified 38 miRNAs that might target *LINC00152* molecules as shown in Figure 2. Of these miRNAs, several miRNA molecules have been reported to promote TB infection development and progression. The most interesting ones seem to be the following four candidate miRNAs: miR-16 [27], miR-125b [28], miR-206 [29], and miR-424 [30]. Indeed, the published literature shows that levels of these four candidate miRNAs are increased in TB patient samples and MTB-infected cells. Our experimental results, bioinformatics predictions, and published articles suggest that *LINC00152* acting as an miRNA sponge might interact with these candidate miRNAs to promote TB development and progression. Our observations can thus guide future studies on the biological mechanisms of *LINC00152* in TB infection.

The management of TB disease is frequently associated with serious complications, including several adverse reactions to ATDs. Liver injury is the most common and serious adverse effect of TB therapy involving isoniazid, rifampicin, and pyrazinamide, which affects 5%-28% of TB patients [31–32]. A better understanding of risk factors of anti-TB induced liver injury might help prevent this condition, which occasionally contributes to the discontinuation of treatment. Some studies have reported several genetic risk biomarkers such as rs1695 in the GSTP1 gene [32–33] and the slow-acetylator genotype of the NAT2 gene [32, 34]. Other studies have mainly focused on drug metabolizing enzyme gene variants as candidate markers of drug-induced liver injury. We are the first to find that *LINC00152* rs80292941 may be a TB predisposition factor and that AA genotype carriers are almost four times more likely to develop RIP- and INH-based anti-TB liver injuries than patients with AT/TT genotypes. Yue *et al* reported that *LINC00152* is involved in xaliplatin (L-OHP) drug resistance in cases of colon carcinoma [23]. These results indicate that *LINC00152* and its variant may be involved in host responses to drug treatment. However, their roles in adverse drug reactions remain unclear, warranting further investigation.

Although our study successfully highlights the significance of *LINC00152* expression and of its polymorphism rs80292941 in susceptibility, clinical disease features, and adverse responses to TB drugs, our study suffers from several limitations. First, only one SNP within *LINC00152* among western Chinese was studied. Future studies should explore more variants in genetically diverse populations. Second, future mechanistic investigations must elucidate specific biological roles of *LINC00152* in TB infection. Third, higher risks of rs80292941 AA genotyping associated with anti-TB drug-related liver injury require further investigation through multi-center studies and functional experimental research.

In conclusion, we report that *LINC00152* expression is significantly downregulated in TB cases and that *LINC00152* rs80292941 may serve as a predisposing factor for anti-TB induced hepatotoxicity. Our findings strongly suggest that *LINC00152* and its variant promote TB infection and progression.

## MATERIALS AND METHODS

### Study subjects

In our prospective observational study, we preliminarily enrolled 884 patients and 475 unrelated healthy controls from the West China Hospital of Sichuan University in western China from Dec. 2012 to Nov. 2015. TB diagnoses were made for 633 patients based on typical symptoms of active TB disease and based on the identification of microbiological and radiological evidence of active TB. All cases of active TB were confirmed by two experienced respiratory physicians. Regimens with antituberculosis drugs (ATDs) were administered for four or more months, according to the clinical practices recommended by the World Health Organization (WHO) [35]. Patients meeting the following criteria were excluded: (a) no microbiological or radiological evidence of TB; (b) signs of hepatotoxicity, renal injury, and hematological toxicity prior to anti-TB chemotherapy; (c) positive serological testing for HIV, HBV, or HCV; (d) history of chronic liver/kidney/hematological system disease; (e) pregnancy; (f) poor adherence to treatment or to one-year follow-up observations. Controls were recruited from healthy blood donors who had negative interferon gamma release assay (IGRA) results, normal physical examination results, and no history of TB.

**Figure 3:**
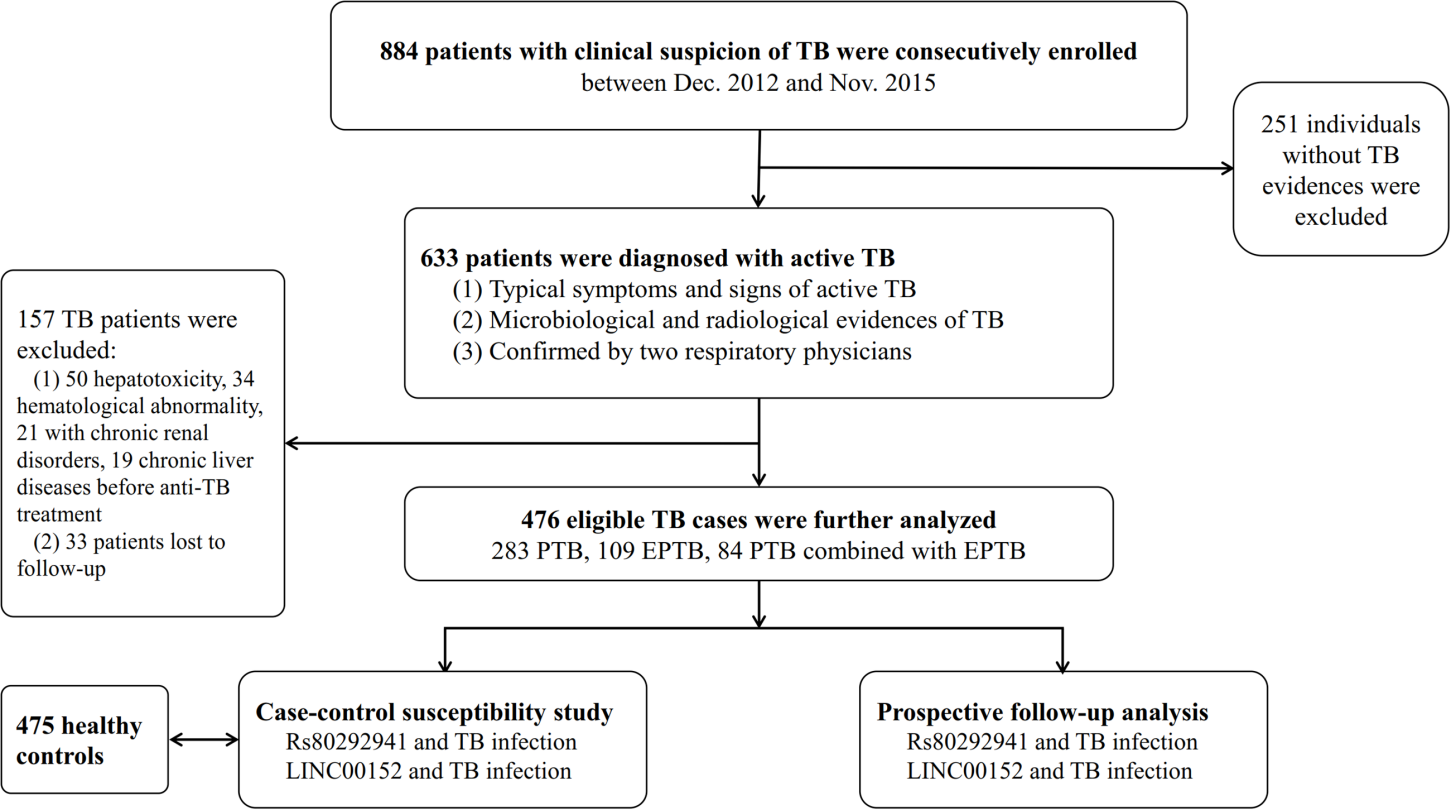
Flow diagram of enrollment of study participants

Demographic data and clinical information of the studied population were reviewed from medical records. Ethylenediaminetetraacetic acid (EDTA)-anticoagulated whole blood samples were collected from each subject for genotyping and lncRNA expression. After starting ATDs, biochemical parameters, hematological blood counts and urinalyses were measured monthly to evaluate adverse reactions to ATDs until the end of the treatment period. Adverse reactions to ATDs covered cases of drug-induced hepatotoxicity, CKD, and hematological toxicity. Drug-induced hepatotoxicity was determined according to criteria on drug-induced liver disorders [36]. Individuals presenting AST and ALT levels of more than twice the maximum limit were considered to have hepatotoxicity. CKD was evaluated as the consensus of drug-induced kidney disease by Mehta *et al*, defined as the persistence of kidney injury (hematuria, proteinuria, casts, and so on) for > 90 days [37]. Anemia (Hb ≤ 90×10^12^/L), leukopenia (absolute counts < 3.5×10^9^/L), and thrombocytopenia (absolute counts < 90×10^9^/L) were considered forms of hematological toxicity [38]. Figure 3 shows a flow diagram of the process used to enroll participants. This hospital-based case-control study was approved of by the Review Board of West China Hospital, Sichuan University (Sichuan, China). All individuals provided written informed consent to participate in the study.

### Single nucleotide polymorphism selection and genotyping

Rs80292941 of *LINC00152* was obtained from the dbSNP (http://www.ncbi.nlm.nih.gov/projects/SNP/.) and lncRNA SNP databases (http://bioinfo.life.hust.edu.cn/lncRNASNP/). It is located at position 87,480,257 in chromosome 2 (GRCh38.p7). Genomic DNA was extracted from blood samples using a QIAamp® DNA blood mini kit (Qiagen, Germany). DNA samples (10 ng) were amplified and genotyped using a custom-by-design 48-Plex SNPscan Kit (Genesky Biotechnologies Inc., Shanghai, China) as described above according to the manufacturer’s recommendations. The commercial kit is based on patented SNP genotyping technology with a double-ligation and multiplex fluorescence PCR developed by Genesky Biotechnologies Inc. For quality control purposes, repeated analyses were carried out for 10% of the randomly selected samples presenting high DNA quality.

### Measurement of *LINC00152* expression

Total RNA was isolated from the peripheral blood mononuclear cells of patients using TRIzol reagent (Invitrogen, CA, USA) and was then converted into complementary DNA (cDNA) using an Omniscript® Reverse Transcription Kit (Qiagen, CA, USA). The RT-qPCR reaction involved 5 μl of SYBR® Premix Ex Taq™ II (Takara, Dalian, China), 1 μl of 10 μM forward and reverse primer (forward: 5′-GCTCCTGGCACAGTCTTTTCTC-3′, reverse: 5′-GGCTGGCAAGTTTCCAATATACA-3′), 3 μl of water and 1 μl of cDNA template. RT-qPCR was performed in a LightCycler® 480 Real-Time PCR System (Roche Diagnostics, NJ, USA) involving an initial denaturation of 15 min at 96°C, amplification for 45 cycles by denaturing at 96°C for 10 s, annealing at 60°C for 30 s and extension at 72°C for 30 s. The samples were denatured at 95°C for 30 s and then heated to 60°C for 30 s at a rate of 0.1°C/s. Fluorescence was measured to generate the melting curve of the amplified products. Raw data were analyzed using Gene Scanning v1.2 software. *LINC00152* expression was normalized to the endogenous control GAPDH and was calculated according to the 2-ΔCT method.

### Statistical analysis

Chi-square and Mann-Whitney U test results were evaluated for categorical and continuous variables. HWE among the controls was determined through Fisher’s exact test. Associations between rs80292941 and *LINC00152* expression, TB risk, and adverse drug reactions were evaluated on the basis of allele frequency distributions/genetic models (additive, dominant and recessive model) and were calculated through a Chi-square test. The strength of association was estimated with odds ratios (ORs) and at a 95% confidence interval (CI). The above listed statistical methods were performed using SPSS version 20.0 (IBM, Chicago, USA). All tests were two-sided, and statistical significance was set at an alpha level of 0.05.

## SUPPLEMENTARY MATERIALS FIGURES



## Abbreviations

ALT: alanine transaminase
AST: aspartate transaminase
ATDs: antituberculosis drugs
ceRNA: competing endogenous RNA
CI: confidence interval
CKD: chronic kidney damage
CRP: C-reactive protein
CT: computed tomography
EMT: epithelial to mesenchymal transition
ENCODE: Encyclopedia of DNA Elements
EPTB: extra-pulmonary tuberculosis
ESCC: early occurrence of esophageal squamous cell carcinoma
ESR: erythrocyte sedimentation rate
GBC: gallbladder cancer
Hb: hemoglobin
HBV: hepatitis B virus
HC: healthy controls
Hct: hematocrit
HIF-1α: hypoxia inducible factor-1α
HIV: human immunodeficiency virus
HWE: Hardy-Weinberg equilibrium
IGRA: interferon gamma release assay
INH: isoniazid
*LINC00152*: long intergenic non-coding RNA 00152
LncRNA: long non-coding RNA
MAF: the minor allele frequency
MDR-TB: multidrug-resistant tuberculosis
MTB: Mycobacterium tuberculosis
ORs: odds ratios
PI3K: phosphatidylinositol 3-kinase
PTB & EPTB: pulmonary tuberculosis combined with extra-pulmonary tuberculosis
PTB: pulmonary tuberculosis
RIF: rifampicin
SNP: single nucleotide polymorphism
SP1: specificity protein 1
TB: tuberculosis
WHO: World Health Organization
