# Supplementary material for: Clinical relevance of LINC00152 and its variants in western Chinese tuberculosis patients
Source: Oncotarget. 2017 Dec 14;8(70):115456–68. doi: 10.18632/oncotarget.23297 (PMC5777785; doi:10.18632/oncotarget.23297)
Supplement: Supplementary file 1 [file oncotarget-08-115456-s001.pdf]

Clinical relevance of *LINC00152* and its variants in western Chinese tuberculosis patients

SUPPLEMENTARY MATERIALS

**miRNA**	hsa-miR-4434	
**SNP**	rs80292941	
**lncRNA**	lnc-PLGLB2-1:10	
**TargetScan**	**start**	**end**
	87,779,775	87,779,781
**miRanda**	**start**	**end**
	87,779,765	87,779,782
	**Score:** 140.0 **Energy:** -12.43 kCal/mol **Strand:** +	
	Q: 2 to 9 R: 15 to 32 Align Len (7) (100.0%) (100.0%)	
	miRNA: 3' aagaugaaauGAAGAGGa 5'<br>||||||| lncRNA: 5' acccagcaacCTTCTCCt 3'	

Supplementary Figure 1: SNP causes miRNA:lncRNA gain.
Note: SNP rs80292941 T allele interacts with has-miR-4434; Lnc-PLGLB2-1:10 represents *LINC00152*.

<table>
<tr><td>miRNA</td><td colspan="2">hsa-miR-1273f</td></tr>
<tr><td>SNP</td><td colspan="2">rs80292941</td></tr>
<tr><td>lncRNA</td><td colspan="2">lnc-PLGLB2-1:10</td></tr>
<tr><td rowspan="2">TargetScan</td><td>start</td><td>end</td></tr>
<tr><td>87,779,774</td><td>87,779,780</td></tr>
<tr><td rowspan="5">miRanda</td><td>start</td><td>end</td></tr>
<tr><td>87,779,763</td><td>87,779,781</td></tr>
<tr><td colspan="2">Score: 150.0 Energy: -21.06 kCal/mol Strand: +</td></tr>
<tr><td colspan="2">Q: 2 to 15 R: 13 to 31 Align Len (13) (76.92%) (84.62%)</td></tr>
<tr><td colspan="2">miRNA: 3' gugacGUUGGAGGUAGAGg 5'
||:| ||x||||
lncRNA: 5' ggaccCAGCAACCATCTCc 3'</td></tr>
</table>

Supplementary Figure 2: SNP causes miRNA:lncRNA loss.
Note: Rs80292941 A allele losses the ability to bind has-miR-1273f; Lnc-PLGLB2-1:10 represents *LINC00152*.
